# Genotype‐By‐Environment Interaction for Scrotal Circumference in Young Nellore Bulls Under Pasture and Feedlot Conditions and Its Correlation With Days to Calving

**DOI:** 10.1002/age.70105

**Published:** 2026-04-16

**Authors:** Christiane A. Silva, Annaiza B. Bignardi, Joslaine N. S. G. Cyrillo, Maria E. Z. Mercadante, Mário L. Santana

**Affiliations:** ^1^ Grupo de Melhoramento Animal de Mato Grosso (GMAT), Instituto de Ciências Agrárias e Tecnológicas Universidade Federal de Rondonópolis (UFR) Rondonópolis MT Brazil; ^2^ Instituto de Zootecnia (IZ) Centro Avançado de Pesquisa de Bovinos de Corte Sertãozinho SP Brazil

**Keywords:** beef cattle, genetic parameters, heritability, random regression, reaction norm, reproduction, sexual precocity

## Abstract

Scrotal circumference (SC) is a key reproductive indicator in beef cattle and an important predictor of male puberty and correlated female fertility. In this study, we evaluated young Nellore bulls raised under combinations of pasture and feedlot environments and modeled SC using a “double” reaction norm model. Additive genetic variance for SC increased from approximately 1.3 cm^2^ under the poorest environmental combinations to about 3.5 cm^2^ under the most favorable conditions, while heritability rose from roughly 0.30–0.33 to 0.60–0.63 across the same gradient. The genetic (co)variance structure indicated that most genetic variation was associated with the intercept, whereas pasture‐ and feedlot‐specific slopes represented smaller yet relevant components of environmental sensitivity. Positive correlations between the intercept and both slopes (0.21 for pasture and 0.50 for feedlot) showed that bulls with higher baseline SC tended to express stronger responses across environments, particularly under feedlot conditions. Conversely, the negative correlation between pasture and feedlot slopes (−0.64) revealed an antagonistic pattern or a trade‐off between sensitivities to these two axes. Genetic correlations for SC across environments were predominantly high and positive (≥ 0.80), declining only under the most divergent combinations. Correlations between SC and days to calving were consistently negative, ranging from −0.051 to −0.112 for approximated estimates and from −0.127 to −0.192 for partial estimates. Overall, the “double” reaction norm approach proved effective for dissecting genotype‐by‐environment interactions and guiding selection strategies aimed at improving reproductive efficiency in tropical beef cattle.

## 1 Introduction

Scrotal circumference (SC) is a key indicator of reproductive development and function in beef cattle and has long been incorporated into breeding objectives for both Zebu and taurine breeds. This trait reflects testicular growth, sperm production potential, and endocrine activity associated with puberty and fertility (Bremer et al. 2023; Butler et al. 2020; Fortes et al. 2013). Selection for larger SC has been widely adopted as an indirect criterion to improve reproductive efficiency, not only because of its moderate to high heritability but also due to its favorable genetic association with semen quality and early sexual maturity in bulls (Carvalho et al. 2023; Silva et al. 2013). From a breeding standpoint, SC represents an easily measurable, low‐cost, and early indicator of reproductive performance, making it particularly valuable in tropical beef cattle improvement programs.

Beyond its direct implications for male fertility, SC is genetically associated with female reproductive performance. Although this association is generally modest, it has been consistently reported as favorable in several beef cattle populations for traits such as age at first calving, calving interval, and days to calving (DC) defined as the interval between the start of the breeding season and the date of calving (Carvalho et al. 2023; Johnston et al. 2014; Santana et al. 2015). Therefore, selection for increased SC in young bulls can indirectly enhance, to some extent, the reproductive efficiency of their female relatives. This association is biologically plausible because SC reflects endocrine function and spermatogenic activity governed by many of the same hormonal pathways that regulate female fertility (de Camargo et al. 2015; Fortes et al. 2013; Utsunomiya et al. 2014). Consequently, understanding the genetic basis and environmental sensitivity of SC and its relationship with female reproductive performance is essential for the coordinated improvement of reproductive efficiency in beef cattle.

Beef production in tropical and subtropical regions is characterized by marked environmental heterogeneity. In countries of the Southern Hemisphere, such as Brazil, Argentina, and Australia, beef cattle are typically raised on extensive pastures during most of their growth phase and subsequently finished in feedlots before slaughter (Ferraz and de Felício 2010). These contrasting production systems differ substantially in nutritional availability, thermal load, and management practices, creating distinct environmental challenges that can modify the expression of growth and reproductive traits. Such environmental contrasts provide a natural framework for the occurrence of genotype‐by‐environment interactions (G × E), in which animals with superior genetic merit in one environment may not maintain the same ranking in another (Silva Neto et al. 2024). Evidence of G × E for growth and reproductive traits has been reported in Nellore cattle under both pasture and feedlot conditions (Raidan et al. 2016; Santana et al. 2025), highlighting the importance of accounting for environmental sensitivity in genetic evaluations and selection programs.

Despite the biological relevance of SC and its established association with female fertility, little is known about how its genetic expression varies across contrasting production environments or how G × E might influence its genetic association with DC. The use of reaction norm models provides a robust framework to address these questions (Santana et al. 2025; Silva Neto et al. 2024). Therefore, the present study aimed: (1) to estimate (co)variance components and genetic parameters for SC in young Nellore bulls across pasture and feedlot environments using a “double” reaction norm model; (2) to quantify the environmental sensitivity of SC along both gradients and characterize the genetic structure underlying animals' responses to contrasting environmental conditions; and (3) to conduct a preliminary assessment of the genetic relationship between SC and female DC. This research provides novel insights into the environmental modulation of SC and its implications for the design of selection strategies in tropical beef cattle populations.

## 2 Materials and Methods

### 2.1 Data

The data analyzed in this study originated from the APTA Beef Cattle Center, Institute of Animal Science, located in Sertãozinho, São Paulo, Brazil (21°10′ S, 48°50′ W). Records from young Nellore males born between 1981 and 2022 were used, together with a pedigree file containing 8636 animals. Calves were reared on pastures composed mainly of *Panicum maximum* and *Brachiaria brizantha* grasses, and weaning was performed at approximately 7 months of age. After weaning, all male calves (averaging approximately 120 per birth year) were moved to feedlot pens of about 3600 m^2^, where they were maintained for a 168‐day growing period. Feed was provided ad libitum twice daily and consisted of corn silage, hay, soybean meal, ground corn, and a mineral supplement containing urea. At the end of the feedlot phase, the males were weighed (W378, in kg) and their SC (in cm) was measured at approximately 378 days of age (range: 300–446 days). In contrast, all female calves remained exclusively under pasture‐based management throughout their development, including during the reproductive phase in which DC was recorded.

The Nellore population analyzed in this study originates from an experimental herd established in 1976, with the objective of improving yearling weight through individual performance selection. In 1980, the herd was structured into three distinct selection lines: Nellore Control (NeC), Nellore Selection (NeS), and Nellore Traditional (NeT). The NeC line has been maintained as a closed control group, in which sires originated exclusively from within the program and animals were selected based on the population mean for yearling weight. In contrast, the NeS and NeT lines have been selected for superior yearling weight differentials to promote genetic gain for growth. Both the NeC and NeS lines remain closed, whereas the NeT line has periodically incorporated sires from the other two lines as well as from commercial herds. Since 2012, the NeT line has also been subjected to selection for lower residual feed intake, reflecting an additional emphasis on feed efficiency. More details about the experimental selection program can be found in Mercadante et al. (2003) and Cardoso et al. (2018).

Contemporary groups (CG) were formed by jointly considering selection line, year, and month of birth. Groups containing fewer than five observations, as well as individual records that deviated by more than 3.5 standard deviations from the phenotypic mean of their respective CG were excluded from the analyses to ensure data consistency. A descriptive summary of the final dataset used for SC is presented in Table 1.

**TABLE 1:** Summary of the data structure for scrotal circumference in young Nellore bulls according to the selection line (Nellore Control—NeC, Nellore Selection—NeS, and Nellore Traditional—NeT).

Item	NeC	NeS	NeT
Number of animals in the pedigree file	1086	2293	3273
Number of animals with phenotypic records	632	1365	1799
Number of genotyped animals	567	1104	1651
Number of genotyped animals with phenotypic records	250	479	822
Number of sires with progeny with genotypic and phenotypic records	76	129	168
Number of dams with progeny with phenotypic records	298	648	819
Number of sires with progeny with genotypic and phenotypic records	130	296	454
Number of contemporary groups	73	93	97
Phenotypic mean of the trait, cm	22.04	23.10	23.83
Standard deviation of the trait, cm	2.36	2.54	2.91
Maximum phenotypic value of the trait, cm	31.00	32.00	34.50
Minimum phenotypic value of the trait, cm	15.60	16.00	15.00

### 2.2 Genotypes

A total of 3350 genotyped individuals were used in this study, including 1551 males with SC record and their relatives. The animals were genotyped using SNP (single nucleotide polymorphism) panels of different densities over the years (Illumina BovineHD BeadChip, San Diego, CA, USA, *n* = 780; GeneSeek Profiler 75 K—Indicine, Lincoln, NE, USA, *n* = 1318; GGP Indicus 50 K, Lincoln, NE, USA, *n* = 1252). Animals genotyped with medium‐density SNP panels were imputed to a high‐density (HD) SNP panel containing ~770 K SNP markers, using a reference population of 6862 animals genotyped with the HD SNP panel and the software FImpute v3 (Sargolzaei et al. 2014).

The criteria considered for genotype quality control were: (1) inclusion of only autosomal SNP with a GenCall score greater than 0.80; (2) removal of SNP with a minor allele frequency ≤ 0.05; (3) removal of SNP and samples with a call rate ≤ 0.90; (4) removal of SNP with extreme deviation from Hardy–Weinberg equilibrium (*p* value ≤ 10^−5^); (5) removal of SNP with duplicated or unknown positions; and (6) removal of SNP or samples with Mendelian conflicts. After the genomic quality control, 383 739 SNP markers and 3350 genotyped animals remained in the database for subsequent analyses.

### 2.3 Environmental Descriptors of Pasture and Feedlot Conditions

To characterize the environmental conditions experienced by the animals under pasture and feedlot management, two quantitative environmental descriptors were adopted as proposed by Santana et al. (2025). The first descriptor was derived from the animals' performance for weaning weight (W210, in kg) recorded at the end of the grazing period, whereas the second was based on W378, measured at the end of the feedlot period. These weight records were selected to derive the environmental descriptors because they were available for all animals in the dataset, while SC measurements were obtained only at the end of the feedlot period. Moreover, body weight and weight gain records have been successfully used in several previous studies of G × E in beef cattle to represent the quality of environmental conditions experienced by the animals (Carvalheiro et al. 2019; Santana et al. 2023; Silva Neto et al. 2024).

Each weight trait (W210 and W378) was analyzed separately using a single‐trait animal model. The models included the effect of CG, the linear covariate of the animal's age at measurement, and the class effect of the dam's age at calving, grouped into categories ranging from 3 to 10 years or older. Random effects included the direct additive genetic effect, the maternal additive genetic and maternal permanent environmental effects (for W210 only), and the residual effect. The solutions for CG (Best Linear Unbiased Estimates, BLUE) obtained from these models were subsequently used as environmental descriptors representing pasture and feedlot quality, respectively. Thus, every animal with a record for SC was assigned two environmental descriptors corresponding to the pasture and feedlot conditions in which it was raised.

Since the environmental descriptors differed in scale and the dataset comprised three distinct selection lines, the values of each environmental gradient descriptor were standardized within line to a mean of 0 and a standard deviation (SD) of 1, following the procedure described by Santana et al. (2025). This within‐line standardization was necessary because animals from the selected lines (NeT and NeS) exhibited higher average performance than those from the control line (NeC). Consequently, their phenotypes resulted in higher CG solutions, which could otherwise lead to the erroneous interpretation that the environments were inherently more favorable for NeT and NeS animals compared with NeC. In practice, however, the three selection lines were managed under essentially similar environmental conditions.

**FIGURE 1:**
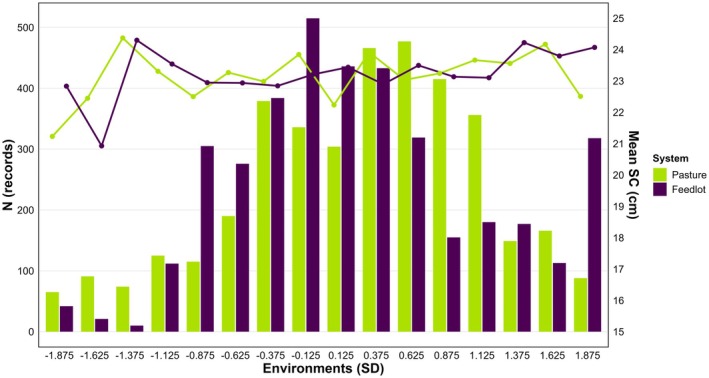
Distribution of records (bars) and mean (lines) scrotal circumference (SC) of young Nellore bulls across pasture and feedlot environments (expressed in standard deviations, SD).

To ensure an adequate number of observations across the environmental gradient for reliable estimation of (co)variance components, the standardized values of each environmental descriptor were grouped into 16 classes, each corresponding to an interval of 0.25 standard deviations (SD), ranging from −1.875 to 1.875. Values > 1.875 or < than −1.875 SD were grouped into the nearest class. This approach aimed to avoid regions of the gradient with very low information, particularly at the extremes, which could compromise the stability of (co)variance component estimates. In addition, SC records were classified according to pasture and feedlot environments to define heterogeneous residual variances across four environmental categories: (1) ≤ 0 SD “poor” pasture and ≤ 0 SD “poor” feedlot; (2) ≤ 0 SD “poor” pasture and > 0 SD “good” feedlot; (3) > 0 SD “good” pasture and ≤ 0 SD “poor” feedlot; and (4) > 0 SD “good” pasture and > 0 SD “good” feedlot. Figure 1 illustrates the distribution of SC records across these environmental combinations.

### 2.4 “Double” Reaction Norm Model for Genetic Evaluation of SC

Given that the primary aim of this study was to evaluate the genetic sensitivity of animals across pasture and feedlot environments, a “double” reaction norm model was applied to estimate (co)variance components and genetic parameters for SC. This approach extends the reaction norm framework by simultaneously modeling two environmental gradients (pasture and feedlot) described in the previous section and initially proposed by Santana et al. (2025). The model was implemented using a single‐step genomic framework, enabling the joint estimation of additive genetic effects across both environmental axes while accounting for the covariance structure between them. The reaction norm model can be described as follows:

$$y=\mathrm{X\beta}+Z_{0}d_{0}+Z_{s-P}d_{s-P}+Z_{s-F}d_{s-F}+e,$$

where y was the vector of observations; β was the vector of systematic effects of CG (as previously defined), age at measurement as a linear covariate, and the class effect of the dam's age at calving (as previously defined, 8 classes); $d_{0}$ was the vector of direct additive genetic intercept of the reaction norms; $d_{s-P}$ was the vector of direct additive genetic slopes of the reaction norms for pasture environments; $d_{s-F}$ was the vector of direct additive genetic slopes of the reaction norms for feedlot environments; and e was the vector of residual effects. A heterogeneous residual variance structure with four classes was adopted as described in the previous section. The incidence matrices X, $Z_{0}$, $Z_{s-P}$, and $Z_{s-F}$ link phenotypic records to their corresponding systematic effects, the direct additive genetic intercept, and the direct additive genetic slopes for pasture and feedlot environments, respectively.

For each animal, the genomic estimated breeding value (GEBV) for SC at a given combination of pasture and feedlot conditions was expressed as follows:

$$a_{i}\left(P,F\right)=f{\left(P,F\right)}^{⊤}\alpha_{i}$$

where $f\left(P,F\right)$ was the vector of environmental covariates and $\alpha_{i}$ was the vector of random regression coefficients for animal i. In the present study, a first‐order (linear) specification was adopted:

$$f\left(P,F\right)=\left[\begin{array}{c} 1\;\\ P\;\\ F \end{array}\right];\alpha_{i}=\left[\begin{array}{c} \alpha_{0i}\;\\ \alpha_{\mathrm{Pi}}\;\\ \alpha_{\mathrm{Fi}} \end{array}\right],$$

where $\alpha_{0i}$ was the intercept (general genetic level for SC); $\alpha_{\mathrm{Pi}}$ was the specific response (slope) to the pasture environment; and $\alpha_{\mathrm{Fi}}$ was the specific response (slope) to the feedlot environment. Assuming $\alpha_{i}∼N\left(0,K_{a}\right)$, the (co)variance matrix of the random regression coefficients was:

$$K_{a}=\left[\begin{array}{ccc} \sigma_{\alpha_{0}}^{2} & \sigma_{\alpha_{0},\alpha_{P}} & \sigma_{\alpha_{0},\alpha_{F}}\\ \sigma_{\alpha_{0},\alpha_{P}} & \sigma_{\alpha_{P}}^{2} & \sigma_{\alpha_{P},\alpha_{F}}\\ \sigma_{\alpha_{0},\alpha_{F}} & \sigma_{\alpha_{P},\alpha_{F}} & \sigma_{\alpha_{F}}^{2} \end{array}\right].$$

Thus, the additive genetic variance of SC at any specific combination $\left(P,F\right)$ was obtained as:

$$\sigma_{A}^{2}\left(P,F\right)=\mathrm{Var}\left(a_{i}\left(P,F\right)\right)=f{\left(P,F\right)}^{⊤}K_{a}f\left(P,F\right).$$

Accordingly, for the grid of pasture‐feedlot environmental combinations used in this study, the additive genetic variance was obtained as:

$$\sigma_{A}^{2}=\mathrm{diag}\left(WK_{a}W^{⊤}\right),$$

where W was the matrix whose rows were the vectors $f\left(P,F\right)$ for all evaluated combinations. Posterior samples of $K_{a}$ obtained via Gibbs sampling were used to derive posterior distributions of $\sigma_{A}^{2}\left(P,F\right)$ across environments. The additive genetic effects were modeled using a combined pedigree and genomic relationship matrix (H) (Aguilar et al. 2010), following the single‐step genomic BLUP framework. The inverse of H was constructed as:

$$H^{-1}=A^{-1}+\left[\begin{array}{cc} 0 & 0\\ 0 & G^{-1}-A_{22}^{-1} \end{array}\right],$$

where $A^{-1}$ was the inverse of the numerator relationship matrix based on pedigree information for all animals, $A_{22}^{-1}$ corresponds to the inverse of the pedigree‐based relationship matrix for genotyped animals, and $G^{-1}$ was the inverse of the genomic relationship matrix.

The heritability of SC across the two environmental gradients was computed as the proportion of the phenotypic variance explained by additive genetic effects for each pasture‐feedlot combination of environments:

$$h^{2}\left(P,F\right)=\frac{\sigma_{A}^{2}\left(P,F\right)}{\sigma_{A}^{2}\left(P,F\right)+\sigma_{E}^{2}\left(P,F\right)},$$

where $\sigma_{E}^{2}\left(P,F\right)$ represents the residual variance for one of the four classes defined previously. The genetic correlation for SC between any two combinations of pasture‐feedlot environments was obtained as:

$$r_{g}\left[\left(P_{1},F_{1}\right),\left(P_{2},F_{2}\right)\right]=\frac{\mathrm{Cov}\left(a_{i}\left(P_{1},F_{1}\right),a_{i}\left(P_{2},F_{2}\right)\right)}{\sqrt{\sigma_{A}^{2}\left(P_{1},F_{1}\right)\sigma_{A}^{2}\left(P_{2},F_{2}\right)}}=\frac{f{\left(P_{1},F_{1}\right)}^{⊤}K_{a}f\left(P_{2},F_{2}\right)}{\sqrt{\left[f{\left(P_{1},F_{1}\right)}^{⊤}K_{a}f\left(P_{1},F_{1}\right)\right]\left[f{\left(P_{2},F_{2}\right)}^{⊤}K_{a}f\left(P_{2},F_{2}\right)\right]}}.$$

The Gibbs sampler was used to draw samples from the conditional posterior distributions of systematic effects, genomic breeding values, and (co)variance components. Systematic effects and breeding values were sampled from their conditional Gaussian distributions, whereas the genetic and residual (co)variances were drawn from inverted Wishart distributions. The Markov chain consisted of 350 000 iterations, with a burn‐in period of 25 000 and a thinning interval of 25, yielding 13 000 retained samples for posterior inference. Convergence was evaluated visually through inspection of trace plots to ensure proper mixing and stationarity of the chains. All analyses were carried out using the BLUPF90 family of programs: RENUMF90, PREGSF90, GIBBSF90+, and POSTGIBBSF90 (Misztal et al. 2014).

### 2.5 Approximate Genetic Correlation Between SC and DC

An initial attempt was made to perform a two‐trait analysis to estimate the genetic correlation between SC and DC. However, convergence difficulties were observed, most likely resulting from the relatively small number of SC records in the current dataset combined with the high complexity of the fitted model. Thus, we obtained approximate genetic correlations between SC and DC based on Pearson correlation coefficients computed among GEBV. In the present population, DC was recorded for all cows that entered the breeding season and was defined as the interval between the start date of the breeding season and the date of the first calving. Records of cows that did not calve were retained in the analyses by assigning a projected DC value, defined as the highest DC record within each contemporary group plus a 21‐day penalty. This trait has been officially evaluated in the Nellore breeding program through a repeatability animal model that accounts for all breeding‐season records of each cow (Mercadante et al. 2003). As mentioned earlier, all females in this herd are raised exclusively under pasture‐based conditions, and consequently DC reflects reproductive performance expressed solely in grazing environments.

GEBV for DC were obtained from the official 2025 genetic evaluation of this Nellore population, based on a single‐step genomic BLUP approach. Pearson correlations were computed at the individual animal level by matching GEBV for SC and DC for the same animals. Specifically, GEBV for DC were retrieved for all animals meeting the accuracy criterion, and then merged with the corresponding GEBV for SC from the present study, including animals without own SC phenotypes but connected through pedigree and(or) genomic information. Only animals with available GEBV for both traits and BIF accuracy ≥ 0.30 (i.e., accuracies approximated from prediction error variance according to Beef Improvement Federation guidelines) were retained in the analyses. Because the genetic association between SC and DC may be partly mediated by growth potential, we also calculated partial genetic correlations, representing the genetic relationship between SC and DC among animals with similar genetic merit for growth. The partial genetic correlation ($r_{\mathrm{xy},z}$) between SC (x) and DC (y), adjusted for the average growth level (z), was computed as:

$$r_{\mathrm{xy},z}=\frac{r_{\mathrm{xy}}-r_{\mathrm{xz}}r_{\mathrm{yz}}}{\sqrt{\left(1-r_{\mathrm{xz}}^{2}\right)\left(1-r_{\mathrm{yz}}^{2}\right)}},$$

where $r_{\mathrm{xy}}$, $r_{\mathrm{xz}}$, and $r_{\mathrm{yz}}$ were the Pearson correlation coefficients between the corresponding variables. The index z was defined as the mean of standardized GEBV for growth traits (birth weight, W210, and W378), with each trait standardized to mean zero and unit variance prior to averaging. In addition, Pearson correlations were also computed between the GEBV for the random regression coefficients of SC (intercept and slopes) and the GEBV for DC.

## 3 Results

### 3.1 Environmental Distribution of Records and Least Squares Means of SC

The data covered a broad range of environmental conditions, with a higher concentration of observations around intermediate levels and fewer records toward the extremes of both gradients (Figure 1). Across the four environmental combinations, defined by the combinations of pasture and feedlot conditions used in the residual variance modeling, the number of records was 1732 (good pasture–good feedlot), 689 (good pasture–poor feedlot), 399 (poor pasture–good feedlot), and 976 (poor pasture–poor feedlot). The least‐squares means of SC varied across these combinations. The lowest mean was observed under the poorest environmental condition (poor pasture–poor feedlot; 22.9 ± 0.08 cm), whereas the highest mean occurred under poor pasture–good feedlot conditions (23.3 ± 0.12 cm). Intermediate values were found for good pasture–good feedlot (23.1 ± 0.06 cm) and good pasture–poor feedlot (22.6 ± 0.09 cm).

### 3.2 Variance Components and Genetic Parameters

The posterior estimates of (co)variance components showed clear contrasts among parameters describing the genetic and residual structure of SC in Nellore cattle (Table 2). The 95% highest posterior density intervals were relatively wide for most of the genetic parameters. The posterior mean of the additive genetic variance for the intercept ($\sigma_{\alpha_{0}}^{2}$ = 1.983) was the largest, while the variances for the pasture ($\sigma_{\alpha_{P}}^{2}$ = 0.260) and feedlot slopes ($\sigma_{\alpha_{F}}^{2}$ = 0.079) were notably smaller, with the pasture slope presenting the greater magnitude. The covariances between the intercept and slopes for pasture ($\sigma_{\alpha_{0},\alpha_{P}}$ = 0.139) and feedlot ($\sigma_{\alpha_{0},\alpha_{F}}$ = 0.183) were positive, whereas the covariance between slopes was negative ($\sigma_{\alpha_{P},\alpha_{F}}$ = −0.096). The posterior mean of the genetic correlation between the intercept and feedlot slope was higher than that between the intercept and pasture slope, while the correlation between slopes indicated a comparatively strong genetic relationship between these terms. Residual variances decreased gradually from less to more favorable environmental combinations, ranging from 2.860 under poor‐poor to 2.084 under good‐good conditions. The ratio between slope and intercept variances was markedly higher for pasture (0.133) than for feedlot (0.040).

**TABLE 2:** Posterior mean, standard deviation (SD), and 95% highest posterior density interval (HPD95%) of (co)variance estimates obtained based on a reaction norm model fitting scrotal circumference in Nellore cattle.

Parameter^a^	Mean	SD	HPD95%
$\sigma_{\alpha_{0}}^{2}$	1.983	0.201	1.666 to 2.320
$\sigma_{\alpha_{P}}^{2}$	0.260	0.110	0.103 to 0.453
$\sigma_{\alpha_{F}}^{2}$	0.079	0.043	0.028 to 0.160
$\sigma_{\alpha_{0},\alpha_{P}}$	0.139	0.103	−0.033 to 0.306
$\sigma_{\alpha_{0},\alpha_{F}}$	0.183	0.095	0.026 to 0.319
$\sigma_{\alpha_{P},\alpha_{F}}$	−0.096	0.059	−0.208 to −0.013
$\sigma_{E}^{2}-P_{\text{poor}}F_{\text{poor}}$	2.860	0.243	2.455 to 3.257
$\sigma_{E}^{2}-P_{\text{poor}}F_{\text{good}}$	2.643	0.328	2.123 to 3.205
$\sigma_{E}^{2}-P_{\text{good}}F_{\text{poor}}$	2.371	0.260	1.949 to 2.804
$\sigma_{E}^{2}-P_{\text{good}}F_{\text{good}}$	2.084	0.171	1.810 to 2.373
$r_{\alpha_{0},\alpha_{P}}$	0.209	0.158	−0.045 to 0.475
$r_{\alpha_{0},\alpha_{F}}$	0.500	0.249	0.067 to 0.850
$r_{\alpha_{P},\alpha_{F}}$	−0.645	0.229	−0.912 to −0.182
$\sigma_{\alpha_{P}}^{2}/\sigma_{\alpha_{0}}^{2}$	0.133	0.059	0.051 to 0.239
$\sigma_{\alpha_{F}}^{2}/\sigma_{\alpha_{0}}^{2}$	0.040	0.022	0.014 to 0.084

*Note:* $\alpha_{0}$, intercept; $\alpha_{F}$, additive genetic slope for feedlot; $\alpha_{P}$, additive genetic slope for pasture; $\sigma^{2}$, variance; E, residual; F, feedlot; P, pasture; r, genetic correlation; σ, covariance.

^a^ “Poor” refers to environmental descriptor values for pasture or feedlot that are equal to or below the average, while “Good” refers to values above the average.

### 3.3 Additive Genetic Variance and Heritability

**FIGURE 2:**
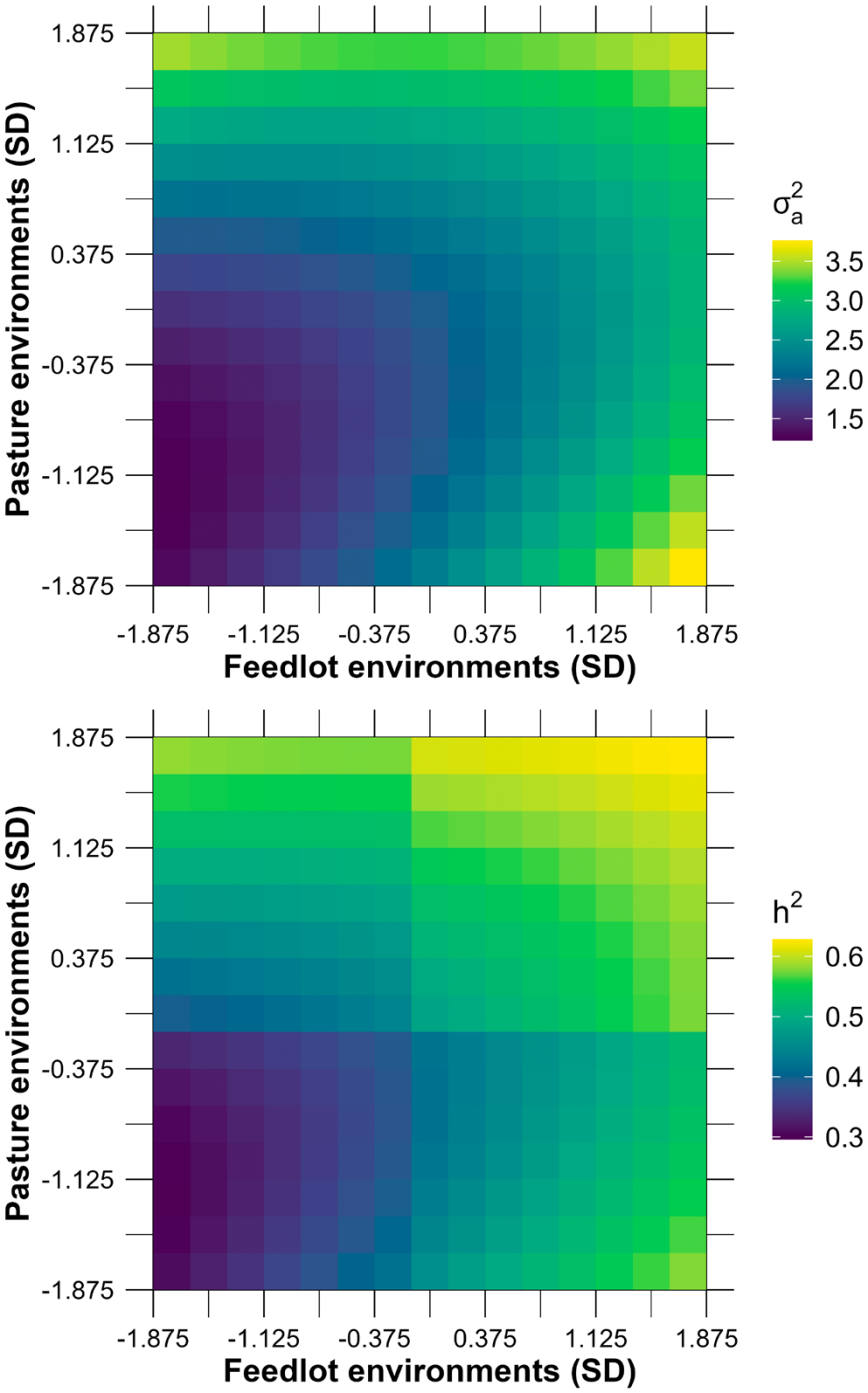
Posterior means of additive genetic variance estimates (top panel) and heritability (bottom panel) for scrotal circumference in young Nellore bulls across pasture and feedlot environments (expressed in standard deviations, SD).

Overall, the joint surface revealed a consistent monotonic increase in additive genetic expression and in the proportion of phenotypic variance attributable to additive effects as environmental quality improved across both pasture and feedlot axes (Figure 2). Both parameters were lowest under the harshest environmental conditions, with additive genetic variance near 1.3 cm^2^ and heritability around 0.30 to 0.33. Values increased steadily with improvements in either axis of the gradient, reaching intermediate levels (additive genetic variance between 2.0 and 2.5 cm^2^; heritability between 0.45 and 0.50) near the center of the environmental space. The highest estimates for both metrics occurred under jointly favorable conditions (Pasture ≥ 1.5; Feedlot ≥ 1.5 SD), where additive genetic variance approached 3.5–3.6 cm^2^ and heritability reached 0.60 to 0.63.

### 3.4 Genetic Correlations for SC Across Pasture‐Feedlot Environmental Combinations

**FIGURE 3:**
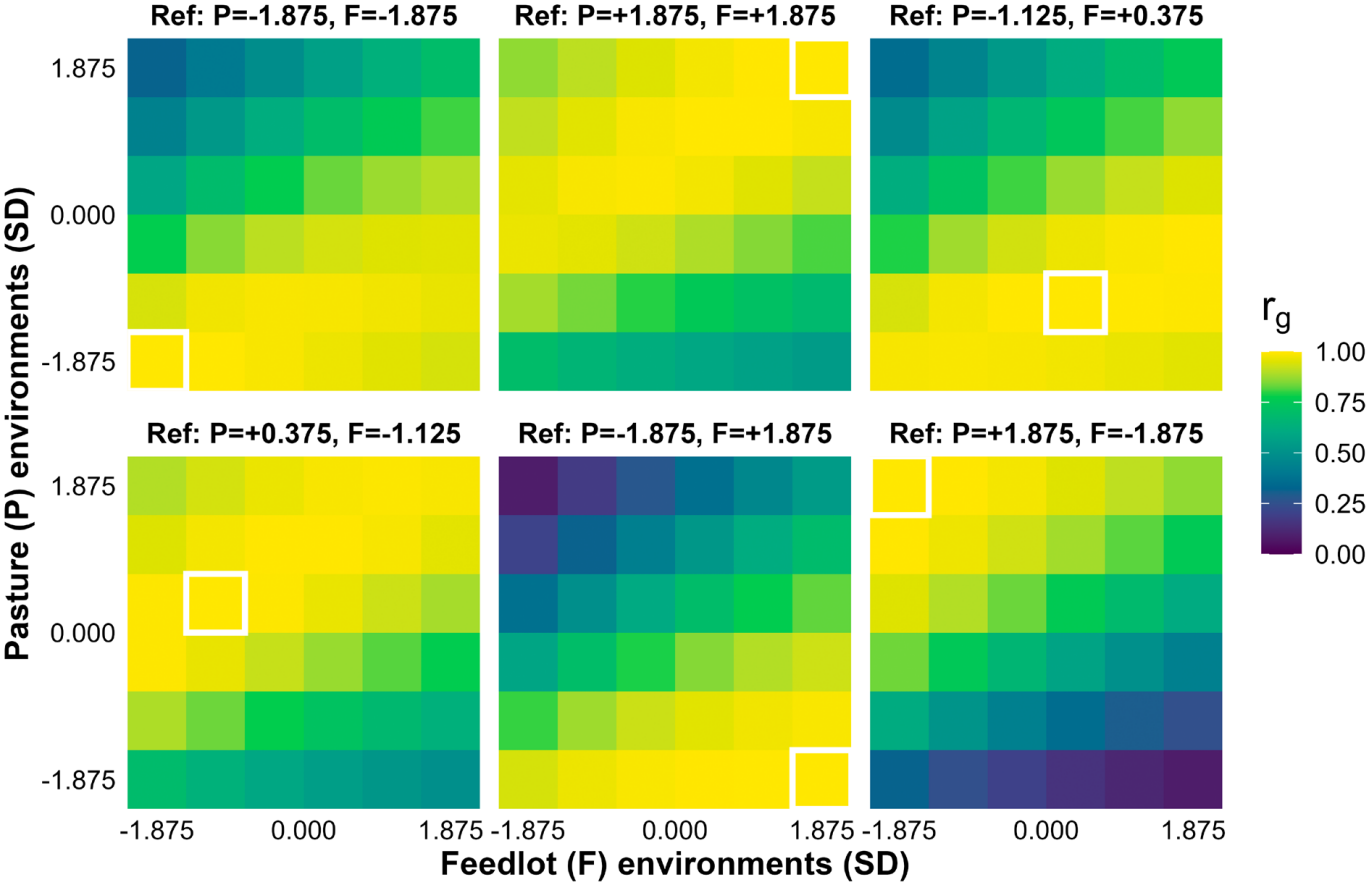
Posterior means of genetic correlations for scrotal circumference in young Nellore bulls across pasture (P) and feedlot (F) environments (expressed in standard deviations, SD). Each panel displays the correlations between a given reference (Ref) environmental combination and all other combinations.

Genetic correlations for SC remained high within similar or neighboring pasture‐feedlot conditions and declined progressively as environmental distance increased (Figure 3). In the extreme reference environments (pasture = −1.875, feedlot = −1.875 and pasture = 1.875, feedlot = 1.875 SD), correlations with nearby combinations were uniformly strong (mostly ≥ 0.9), but decreased to moderate levels (0.6 to 0.7) when contrasted with the opposite corner of the environmental space. Intermediate reference conditions (e.g., pasture = −1.125, feedlot = 0.375 and pasture = 0.375, feedlot = −1.125 SD) showed consistently high correlations (≥ 0.8) with most combinations in the central portion of the gradient, but dropped to approximately 0.4–0.6 when compared with the most distant environmental combinations. In contrast, the most divergent combinations (pasture = −1.875, feedlot = 1.875 and pasture = 1.875, feedlot = −1.875 SD) displayed the steepest decay, with correlations remaining high within their own quadrant but falling to very low values (< 0.2) when compared directly with each other.

### 3.5 Genetic Correlations Between SC and DC

**FIGURE 4:**
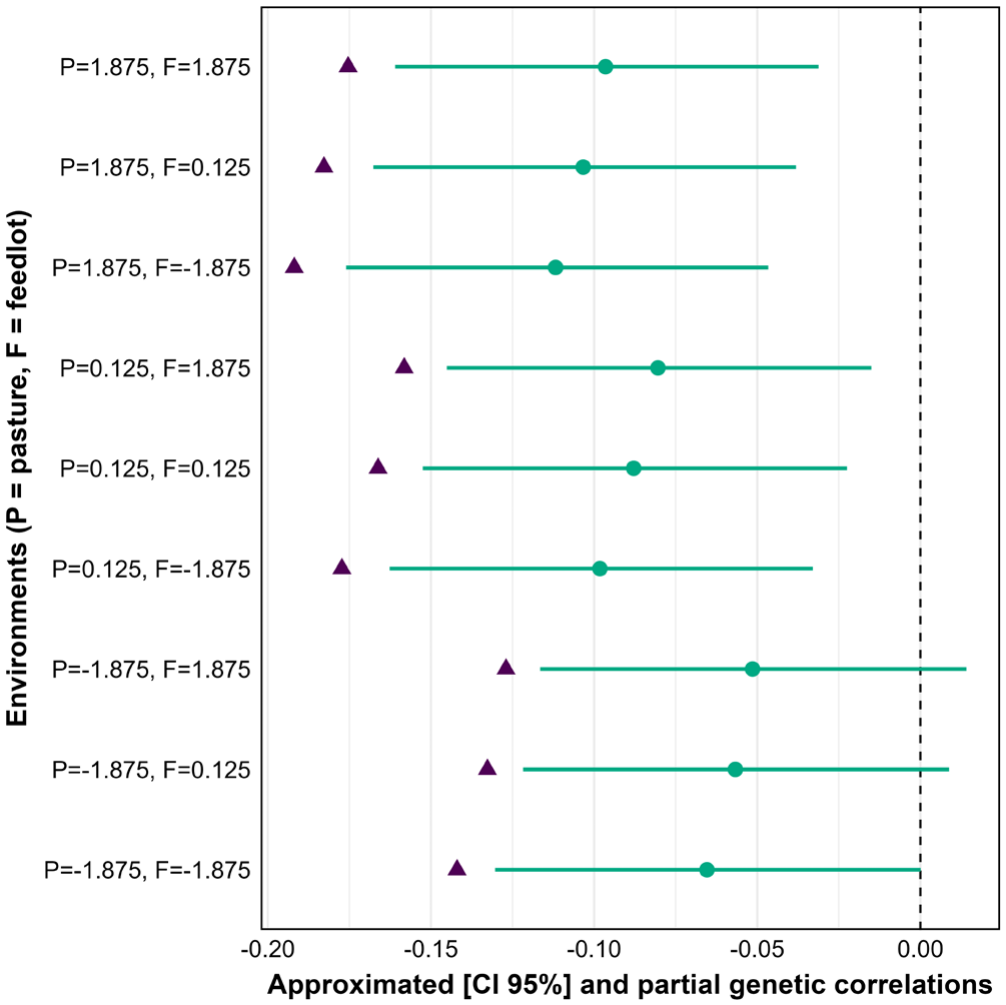
Approximated genetic correlations (circles indicating point estimates with horizontal 95% confidence intervals) and partial genetic correlations (triangles) between scrotal circumference in young Nellore bulls and days to calving across pasture and feedlot environments (expressed in standard deviations).

The approximated genetic correlations (95% frequentist confidence intervals) between the GEBV of the reaction norm terms for intercept, pasture slope, and feedlot slope of SC and DC were −0.087 (−0.152 to −0.021), −0.134 (−0.198 to −0.069), and −0.022 (−0.087 to 0.043), respectively. Only the approximated genetic correlation between the GEBV of the feedlot slope for SC and GEBV for DC was not significantly different from zero (*p* > 0.01). Both the approximate and partial genetic correlations between SC and DC were consistently negative across all pasture‐feedlot environmental combinations (Figure 4). Under the harshest environmental combination (pasture = −1.875; feedlot = −1.875), the approximated correlation between SC and DC was −0.065, whereas the partial correlation adjusted for growth reached −0.142. When pasture was kept at −1.875 and feedlot conditions improved to 0.125 and 1.875, the approximated correlations changed to −0.057 and −0.051, respectively, while the corresponding partial estimates were −0.133 and −0.127. At the intermediate pasture level (0.125), the values ranged from −0.098 (feedlot = −1.875) to −0.080 (feedlot = 1.875) for the approximated correlations, and from −0.177 to −0.158 for the partial correlations. Under the most favorable pasture conditions (1.875), the approximated correlations varied from −0.112 (feedlot = −1.875) to −0.096 (feedlot = 1.875). For these same combinations, the partial correlations ranged from −0.192 to −0.175.

## 4 Discussion

Understanding how environmental heterogeneity shapes phenotypic expression is essential for accurately characterizing genetic variation and optimizing selection strategies in beef cattle populations. Reaction norm models allow the partitioning of genetic and environmental contributions across continuous environmental gradients, providing a framework to detect G × E, quantify environmental sensitivity, and improve the accuracy of breeding value predictions under variable production conditions (Carvalheiro et al. 2019; Mulder 2016).

The least squares means illustrated how SC responds to discrete combinations of pasture and feedlot quality. The lowest mean occurred under the poorest overall conditions, whereas the highest mean was observed when pasture quality was poor, but feedlot conditions were favorable. This pattern is consistent with physiological expectations, because cattle exposed to suboptimal grazing conditions and later provided with improved nutritional supply in feedlot may express elements of compensatory growth. This is a well‐documented phenomenon in which animals accelerate tissue deposition following a period of nutritional restriction, including effects on testicular development and scrotal traits (Fan et al. 2018; Kamalzadeh et al. 1998). The SC development is sensitive to energy balance, protein availability, and endocrine activity associated with pubertal progression (Barth et al. 2008; Brito et al. 2007). However, because SC was measured at the end of the feedlot period, the observed differences primarily reflect conditions during this final phase. Thus, pasture effects are likely indirect, acting through prior growth and physiological status rather than directly on SC development. Although the observed pattern and the negative association between environmental sensitivities are consistent with compensatory responses, the reaction norm model captures genetic responsiveness across environments and does not support causal inference regarding the mechanisms linking pasture conditions, body growth, and SC development.

The additive genetic variance for the intercept showed the greatest magnitude, indicating substantial genetic variability in overall performance of the SC, whereas the variances associated with pasture and feedlot slopes were considerably smaller. This pattern reflects the common structure of random regression models in which most genetic variation is partitioned into overall performance level, with smaller proportions assigned to environmental sensitivity terms (Carvalho Filho et al. 2022; Chiaia et al. 2015; Santana et al. 2015). Among the slope components, the pasture gradient exhibited the larger variance, suggesting greater genetic heterogeneity in the response of SC to pasture conditions. Positive covariances between the intercept and the slope terms indicate that genetically superior animals for baseline SC also tend to express favorable responses along both environmental axes. In contrast, the negative covariance between the two slopes suggests an antagonism or trade‐off between sensitivities to the pasture and feedlot gradients. Although the 95% highest posterior density intervals were relatively wide, the consistency in signs and relative magnitudes supports this general pattern. Based on the stronger genetic relationship between the intercept and the feedlot slope compared with that between the intercept and the pasture slope, selection for higher overall SC levels is expected to result in a more pronounced increase in environmental sensitivity to feedlot conditions. An increase in environmental sensitivity driven by selection for higher performance levels has been previously documented in this population by Freitas et al. (2021) and Santana et al. (2025). Although the present herd includes a relatively small number of animals, nearly five decades of intense selection have contributed to its widespread use as a genetic resource by multiple herds across different regions of Brazil. The genetic material from this herd is extensively disseminated through regular auctions of bulls and cows and, more importantly, through the commercialization of semen in artificial insemination centers, reaching herds managed under diverse production systems. Therefore, the results reported here may have relevant implications for Nellore populations raised under pasture, feedlot systems, or a combination of both.

Residual variances declined steadily from poor to favorable environments, a pattern consistent with reduced environmental noise under improved nutritional and management conditions. This result aligns with findings reported by Raidan et al. (2016, 2017) for SC in young Nellore bulls evaluated in performance tests, where the authors observed posterior residual variances ranging from 2.98 to 3.05 cm^2^ under pasture conditions and from 2.36 to 2.49 cm^2^ under feedlot conditions.

The joint surface of additive genetic variance and heritability reinforced the trends described above by showing a monotonic increase in both parameters as environmental quality improved along the pasture and feedlot gradients. The lowest values were observed under the harshest conditions, where limited nutritional availability and greater environmental stress likely constrained phenotypic expression and reduced the proportion of variance attributable to additive effects. Although the present study was conducted within a single herd managed under relatively favorable conditions compared with many Nellore herds across Brazil, substantial environmental variation remains inherent to more than 40 years of SC records analyzed here. These findings were also consistent with broader evidence from domestic cattle. Schou et al. (2020) showed that microenvironmental variance, approximated by residual variance, tends to decline in more optimal environments for traits exposed to long‐term directional selection, reflecting both improved environmental uniformity and a historical increase in performance. This framework supports the pattern observed here, in which favorable environments appear to allow a more complete expression of the animals' genetic potential. Our results were also aligned with those reported by Raidan et al. (2016, 2017), who found lower posterior mean heritability estimates for SC in young Nellore bulls under pasture‐based performance testing (0.52–0.56) and higher estimates under feedlot conditions (0.60–0.63). In terms of magnitude, our estimates were further comparable to those obtained by Mota et al. (2020) using a reaction norm model for SC records in Nellore cattle, with heritability values ranging from 0.28 in more restrictive environments to 0.56 in more favorable ones.

Genetic correlations for SC across contrasting environments were consistently positive and generally high, indicating that most genes underlying testicular development remain similarly expressed across pasture‐ and feedlot‐based conditions. Chiaia et al. (2015) reported correlations ranging from 0.73 to 1 when SC was evaluated across an environmental gradient, suggesting limited re‐ranking of animals except when environments were highly divergent. Supporting this pattern, Raidan et al. (2016) observed posterior mean correlations of 0.78 to 0.87 for SC measured on pasture versus feedlot, while Raidan et al. (2017) reported similarly strong associations, with genetic correlations between SC on pasture and feedlot averaging 0.84 (highest posterior density interval 0.74–0.93). The SC has previously been described as a relatively robust trait with respect to G × E, with meaningful re‐ranking occurring only under pronounced differences in environmental quality (Santana et al. 2023). Therefore, selection for SC is expected to generate favorable and consistent genetic responses across a wide range of pasture and feedlot environments.

The genetic correlations between SC and DC obtained were consistently negative, indicating that sires with greater genetic potential for testicular development tend to produce daughters that conceive earlier and require fewer days to calving. This pattern was consistent with previous findings in beef cattle. Johnston et al. (2014) reported correlations of −0.15 between SC and DC and −0.21 between SC and heifer pregnancy in Brahman cattle in Australia, while Pereira et al. (2000) and Forni and Albuquerque (2005) documented similarly favorable, though modest, correlations in Nellore females (−0.04 to −0.22 and −0.10, respectively). Comparable results were also reported by Chiaia et al. (2015) in Nellore cattle, who evaluated SC and age at first calving across a postweaning weight gain environmental gradient and found genetic correlations ranging approximately from −0.10 to −0.25, with stronger (more negative) values emerging under favorable environmental conditions. A similar pattern was described by Santana et al. (2023), who reported negative genetic correlations between SC and age at first calving (−0.05 to −0.14) across prenatal environmental conditions in a large Nellore population. More pronounced negative values in more favorable prenatal environments indicated that improved developmental environments seem to amplify the genetic association between male and female reproductive traits. This directional strengthening of the SC‐female reproduction relationship in improved environments mirrors the pattern observed in the present study across the pasture‐feedlot gradient. It is also important to note that DC in this population was recorded exclusively under grazing conditions, which suggests that environmental modulation of the SC‐DC relationship was driven primarily by the pasture axis, whereas improvements in the feedlot axis had little influence. Consistent with this pattern, we found that the genetic correlation between DC and the GEBV for the pasture slope of SC was stronger and more favorable, whereas the correlation with the feedlot slope was close to zero. Therefore, in this population, selection for SC based on overall performance, particularly when considering the response expressed under grazing environments, is expected to produce more favorable correlated responses in DC. These results reinforce the consistency of SC, albeit with modest magnitude, as an indicator of reproductive performance. It is important to note that correlations between GEBV may be affected by estimation error, potentially leading to attenuation of the true genetic association. To mitigate this effect, a minimum accuracy threshold was applied, following a conservative approach as discussed in Saccenti et al. (2020).

The underlying biological basis of the SC‐DC relationship may involve growth‐related pathways, as body weight and growth trajectory show moderate genetic correlations with SC (0.19 to 0.40) (Boligon et al. 2010; Frizzas et al. 2009; Santana et al. 2013) and with reproductive performance of Nellore females (0.22–0.31) (Boligon et al. 2010; Boligon and Albuquerque 2011; Eler et al. 2014). However, the consistently stronger partial correlations after adjusting for weight demonstrate that the SC‐DC relationship extends beyond shared genetic control of growth, pointing to direct pleiotropic mechanisms linking male and female reproductive biology. Such mechanisms are supported by Irano et al. (2016), who identified genomic regions jointly associated with SC and early pregnancy in Nellore cattle, highlighting loci that influence both testicular development and sexual precocity in females. This framework is strengthened by findings from Carvalho Filho et al. (2024), who reported candidate genes and quantitative trait loci with environmentally dependent effects on SC and age at first calving, involving pathways related to lipid metabolism, mitogen‐activated protein kinase signaling, and cellular energy homeostasis. Furthermore, de Carvalho et al. (2025) identified X‐linked genomic regions harboring genes such as TMSB4X, TLR7, and PRPS2 that were associated with both SC and female reproductive performance, reinforcing the existence of shared molecular mechanisms that integrate male testicular development with key aspects of female fertility in Nellore cattle. From a breeding perspective, the persistence of negative partial correlations between SC and DC across all pasture‐feedlot combinations underscores the utility of SC in multi‐trait selection indices aimed at improving reproductive efficiency in Nellore cattle.

## 5 Conclusion

This study characterized the genetic architecture of SC in young Nellore bulls across pasture and feedlot environments. Additive genetic variance and heritability increased toward more favorable conditions, indicating enhanced expression of genetic potential under improved management. Most genetic variation was associated with overall SC level, with smaller but meaningful components reflecting environmental sensitivity. Bulls with higher baseline SC tended to respond more favorably across environments, whereas the negative association between pasture and feedlot sensitivities suggested a trade‐off between these axes. Genetic correlations across environments were high, indicating limited re‐ranking except under highly divergent conditions. The genetic association between SC and DC was consistently favorable and stronger under pasture conditions, where DC was expressed. These findings support the use of a “double” reaction norm approach to refine selection strategies and improve reproductive efficiency in tropical beef production systems.

## Author Contributions

**Christiane A. Silva:** investigation; writing – original draft; writing – review and editing; formal analysis. **Annaiza B. Bignardi:** supervision; visualization; validation; writing – original draft; writing – review and editing. **Joslaine N. S. G. Cyrillo:** data curation; resources; writing – review and editing; **Maria E. Z. Mercadante:** data curation; resources; writing – review and editing. **Mário L. Santana:** conceptualization; investigation; methodology; formal analysis; resources; supervision; writing – original draft; writing – review and editing.

## Funding

The authors have nothing to report.

## Ethics Statement

The farm where the data was collected followed all animal welfare guidelines established by the law No. 11.977 from the state of São Paulo, Brazil. All the datasets analyzed were obtained from an existing database and approval of the Ethics Committee was therefore not necessary.

## Conflicts of Interest

The authors declare no conflicts of interest.

## Data Availability

The data can be requested by contacting JNSGC (jgcyrillo@sp.gov.br) and MEZM (mezmercadante@gmail.com) upon a reasonable request for research purposes and with permission of the experimental breeding program (Nelore‐IZ).
